# Prenatal diagnosis of 21 fetuses with balanced chromosomal abnormalities (BCAs) using whole-genome sequencing

**DOI:** 10.3389/fgene.2022.951829

**Published:** 2022-09-15

**Authors:** Fang Fu, Ru Li, Xiao Dang, Qiuxia Yu, Ke Xu, Weiyue Gu, Dan Wang, Xin Yang, Min Pan, Li Zhen, Yongling Zhang, Fatao Li, Xiangyi Jing, Fucheng Li, Dongzhi Li, Can Liao

**Affiliations:** ^1^ Prenatal Diagnostic Center, Guangzhou Women and Children’s Medical Center, Guangzhou, China; ^2^ Guangzhou Medical University, Guangzhou, China; ^3^ Chigene (Beijing) Translational Medical Research Center Co,. Ltd., Beijing, China

**Keywords:** Balanced chromosomal abnormalities, prenatal diagnosis, whole-genome sequencing, *CACNA1E*, PDCL

## Abstract

Balanced chromosomal abnormalities (BCAs) are the most common chromosomal abnormalities and the frequency of congenital abnormalities is approximately twice as high in newborns with a *de novo* BCA, but a prenatal diagnosis based on BCAs is subject to evaluation. To detect translocation breakpoints and conduct a prenatal diagnosis, we performed whole-genome sequencing (WGS) in 21 subjects who were found BCAs, 19 balanced chromosome translocations and two inversions, in prenatal screening. In 16 BCAs on non-N-masked regions (non-NMRs), WGS detected 13 (81.2%, 13/16) BCAs, including all the inversions. All the breakpoints of 12 (12/14) cases of sufficient DNA were confirmed by Sanger sequencing. In 13 interrupted genes, *CACNA1E* (in case 12) and *STARD7* (in case 17) are known causative and *PDCL* was found in subject (case 11) with situs inversus for the first time. Case 12 with abnormal ultrasound reached a definitive genetic diagnosis of *CACNA1E*-disease, while *STARD7* exon deletion has never been found causative in patients. WGS provides the possibility of prenatal diagnosis in fetuses with BCAs, and its clinical significance also lies in providing data for postnatal diagnosis.

## Introduction

Balanced chromosomal abnormalities (BCAs) can be categorized into reciprocal translocations, changes in either localization or orientation between the two non-homologous chromosomes, inversions in the same chromosome, and Robertsonian translocations, a result of the fusion of two acrocentric chromosomes 13, 14, 15, 21 or 22. BCAs are the most common chromosomal abnormalities in both healthy and diseased individuals and the estimated frequency of all balanced structural rearrangements in the general population is 0.2%–0.4% (Jacobs et al., 1992; Blake et al., 2014). The frequency of congenital abnormalities, e.g., multiple congenital anomalies or intellectual disability (ID), is approximately twice as high in newborns with a *de novo* BCA (6.1% for reciprocal translocation, 9.4% for inversions, and 3.7% for Robertsonian translocations) compared with the risk in the general population. (Warburton, 1991). This suggests a causative link between BCAs and clinical phenotype.

The consequences of *de novo* BCAs prenatally diagnosed are unpredictable, which introduce uncertainty into pathogenicity reports and genetic counseling decisions. The clinical phenotype in newborns with BCAs can be caused by disruption of specific gene(s) at a translocation or inversion breakpoint(s), therefore, characterization of the breakpoints in BCAs has been a key to elucidate the potential Mendelian disorders. (Gianfrancesco et al., 2008; Moller et al., 2008; Kalscheuer et al., 2009). Therefore, prenatal diagnosis of BCAs relies on a high-resolution method of genomic structural variations (SVs) detection, while chromosomal microarray analysis (CMA), the method commonly used in copy number variants (CNVs) testing, cannot detect balanced translocations.

Recently, short-read sequencing-based whole-genome sequencing (WGS) has emerged as a comprehensive diagnostic tool with a continuously improving cost-effectiveness. Redin et al. (2017) performed WGS in 273 subjects with BCAs and congenital abnormalities and revealed that WGS could revise 93% of karyotyping results by at least one sub-band, who also determined that in BCAs, 33.9% resulted in gene disruptions, 5.2% were associated with pathogenic genomic imbalances, and 7.3% disrupted topologically associated domains (TADs). (Redin et al., 2017). Using WGS, Yu et al. (2020) identified BCA breakpoints in 3 cases and reached definitive genetic diagnosis in 4 cases, out of 10 cases.

The aim of this study was to assess the clinical utility of WGS in the prenatal setting, and to explore the potential implications on the decision-making process during perinatal period.

## Materials and Methods

### Study settings and participants

This study was a single center pilot cohort study performed at the Department of Prenatal Diagnostic Centre, Guangzhou Medical University (China) between January 2019 and December 2021, including all cases of BCA(s), not Robertsonian translocation, prenatally suspected after a dedicated prenatal karyotyping. The study was approved by the ethics committee of the Guangzhou Women and Children’s Medical Center and adheres to the ethical standards laid down in the 1964 Declaration of Helsinki and its later amendments. (2019356B-01). All individuals enrolled in this study from Guangzhou Women and Children’s Medical Center and informed consent forms were signed by the participants.

### Whole-genome sequencing

Genomic DNA was extracted from amniotic fluid according to standard protocols and purified using a QIAamp DNA Blood Maxi Kit (Qiagen, Hilden, Germany). Genomic DNA from each sample was sheared as input material for library construction, and the fragmented DNA was further processed with end repair, A-tailing, adapter ligation, and PCR amplification following the manufacturer’s recommended protocols. High-throughput sequencing was performed on NovaSeq6000 (Illumina, United States). A minimal read depth was 30-fold for WGS. Quality control for the paired-end reads was assessed using FastQC (https://www.bioinformatics.babraham.ac.uk/projects/fastqc/) and fastp(Chen et al., 2018) was used for adapter trimming and quality filtering.

### Bioinformatic pipeline and data analysis

Sequencing reads were aligned to the human reference genome GRCh37/UCSC hg19 using BWA-MEM (Li and Durbin, 2010), then SAMtools (Li et al., 2009) and Picard (https://broadinstitute.github.io/picard/) was performed to sort and mark duplicated reads, respectively. SVs were screened using MANTA (Chen et al., 2016) and LUMPY (Layer et al., 2014), and CNVnator (Abyzov et al., 2011) and AMYCNE (Eisfeldt et al., 2018) software packages were used to detect CNVs. Repeat expansions at known loci were called by ExpansionHunter (Dolzhenko et al., 2019).

SVs and CNVs were annotated and ranked by AnnotSV (Geoffroy et al., 2018), they were evaluated by comparison with literature values and databases, such as the Database of Genomic Variants (DGV, http://dgv.tcag.ca/dgv/app/home), the Database of genomic variation and Phenotype in Humans Using Ensembl Resources (DECIPHER, https://www.deciphergenomics.org/), ClinGen Dosage Sensitivity Map (ClinGen, https://www.clinicalgenome.org/) and PubMed (https://pubmed.ncbi.nlm.nih.gov/). According to the guidelines of the American College of Medical Genetics and Genomics (ACMG) and the Clinical Genome Resource (ClinGen) (Richards et al., 2015; Abou Tayoun et al., 2018; Brandt et al., 2020; Riggs et al., 2020), the clinical significance of all identified variations was interpreted and classified into five categories: pathogenic (P), likely pathogenic (LP), variant of uncertain significance (VUS), likely benign, and benign. To complement the uncertainty in the interpretation of CNV breakpoints in the current ACMG guidelines(Brandt et al., 2020; Riggs et al., 2020), we performed extra analysis of the genes in which those breakpoints are located. The clinical phenotypes of patients were extracted to specific Human Phenotype Ontology (HPO, https://hpo.jax.org/) terms to facilitate identifying disease-causing variants. Screening and prioritizing clinically relevant variants on the basis of variants quality, allele frequency, functional impact, inheritance model or familial segregation, available information in published literature, and agreement to explain the patient clinical phenotype. The size of aberration, gene disruptions as well as functional elements like enhancers and regulatory elements in topologically associated domain (TAD) (Wang et al., 2018) regions were used for further interpretation for SVs.

### Validation of variants

PCR amplification and Sanger sequencing of the junction fragments were performed to validate SVs using standard protocols. In brief, PCR products were purified by exonuclease I and sequenced on an ABI 3730 DNA Analyzer using BigDye Terminator v3.1 (Applied Biosystems, United States). Sanger sequencing was applied across the WGS detected breakpoints to generate a unique junction fragment sequence of SVs. Except for insufficient DNA in case 1 and 20, the information of Sanger sequencing and the primers in other 11 subjects with gene interruption were shown in Supplementary Material S1.

## Results

### Study participants

**FIGURE 1:**
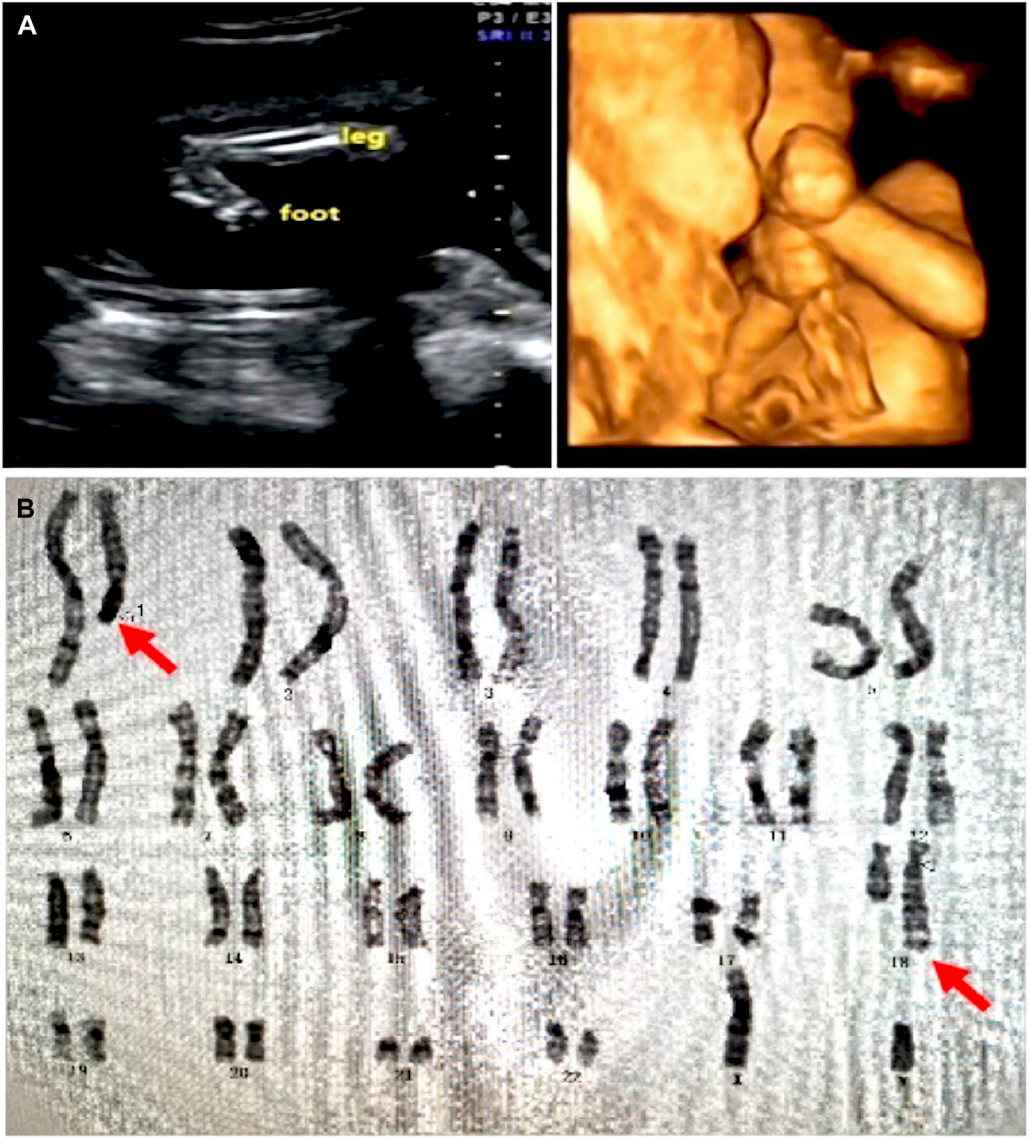
Ultrasound and karyotyping results in case 12. **(A)** Ultrasound showed significant macrocephaly, bilateral talipes calcaneovalgus, and clenching hand. **(B)** Karyotyping showed a balanced translocation 46,XN,t(1; 18) (q25; q23)dn. The original report of karyogram was unavailable. Red arrows showed the mutually translocated chromosomal fragments.

In the 21 subjects, 19 were balanced chromosome translocations and two were inversions. All the subjects had no significant pathogenic genomic variation revealed by previous prenatal screening, including trio karyotyping with a band resolution of 400–650 bands per haploid chromosome set and/or cell-free fetal DNA testing in each subject. Two fetuses were found abnormality by ultrasonography screening (Table 1), in which significant macrocephaly and extremities anomaly were found in case 12 (Figure 1A).

**TABLE 1:** Prenatal findings and the parents’ decision after genetic counseling based on the genetic tests in 21 cases.

Case	Prenatal screening	Ultrasonography screening	Karyotyping	WGS (GRCh37)	Sanger sequencing (GRCh37)	Bias (bp)	Disrupted gene	OMIM phenotype	TADs	Pregnancy outcome
1	AMA	—	46,XY,t(7; 13) (p13; q12.3)dn	chr7:38922939(p14.1)/chr13:35,308,429(q13.2)	Insufficient DNA specimen		VPS4	NA	—	Continued
2	ARH	—	46,XN,t(4; 16) (q34; q21)dn	chr4:187,406,088(q35.2)/chr16:69,409,469(q22.1)	chr4:187,406,088/chr16:69,409,469	0	TERF2	NA	4:187,400,001–187,520,000	Continued
3	HRT21	—	46,XN,t(2; 22) (q11.2; p13)dn	NMR	NA		NA	NA	—	Continued
4	HRT21	—	46,XN,t(1; 12) (q23.1; q15)dn	ND	NA		NA	NA	—	Continued
5	HRT18	—	46,XN,t(6; 16) (p23; q13)dn	ND	NA		NA	NA	—	Continued
6	HRT21	—	46,XX,t(2; 20) (q33; p12.2)dn	ND	NA		NA	NA	—	Continued
7	HRT21	—	46,XY,inv(12) (p11.2q24.1)dn	chr12:31,046,913(p11.21)/chr12:112,346,346(q24.13)	chr12:31,046,916/112,346,346	3	NA	NA	12:31,040,001–31,480,000	Continued
									12:31,040,001–33,000,000	
									12:31,040,001–34,240,000	
									12:1,12,320,001–113,000,000	
8	SCA	—	46,XN,t(1; 4) (?q32.3; p14)dn	chr1:216,632,855(q41)/chr4:39,567,802(p14)	chr1:216,632,855/chr4:39,567,806	4	SMIMI14	NA	—	Continued
9	AA	—	46,XY,t(5; 21) (p15.1; p11.2)dn	NMR	NA		NA	NA	—	Continued
10	AMA	—	46,XX,t(11; 22) (q25; q13.1)dn	NMR	NA		NA	NA	—	Continued
11	—	Situs inversus	46,XX,t(5; 9) (p15.1; q32)dn	chr5:4,049,550(p15.33)/chr9:125,582,738(q33.2)	chr5:4,049,550/chr9:125,582,738	0	PDCL	NA	5:4,040,001–4880,000	Continued
12	—	Macrocephaly with bilateral cerebral ventriculomegaly(12/12 mm), bilateral talipes calcaneovalgus, and clenching hand	46,XN,t(1; 18) (q25; q23)dn	chr1:181,523,009(q25.3)/chr18:73,066,267(q22.3)	chr1:181,523,013/chr18:73,066,267	4	CACNA1E	618,285(Developmental and epileptic encephalopathy 69)	18:73,040,001–74,080,000	Terminated
13	HRT21		46,XN,t(9; 19) (p11; p12)dn	NMR	NA		NA	NA	—	Continued
14	HRT21	—	46,XN,t(7; 18) (q22; q22)pat	NMR	NA		NA	NA	—	Continued
15	ARH	—	46,XN,t(9; 10) (q32; p13)mat	chr9:127,657,832(q33.3)/chr10:3,710,364(p15.2)	chr9:127,657,836/chr10:3,710,364	4	GOLGA1	NA	—	Continued
16	AMA	—	46,XN,t(1; 5) (p31; q11)mat	chr1:68,080,957(p31.3)/chr5:74,423,474(q13.3)	chr1:68,080,957/chr5:74,423,474	0	ANKRD31	NA	5:74,400,001–75,440,000	Continued
17	HRT21	—	46,XN,t(2; 21) (q11.1; q22.3)pat	chr2:96,856,473(q11.2)/chr21:43,781,380(q22.3)	chr2:96,856,473/chr21:43,781,387	7	STARD7	607,876(Epilepsy, familial adult myoclonic, 2)	2:96,840,001 –98,200,000	Continued
18	AMA	—	46,XN,inv(12) (p12.2p13.3)mat	chr12:405,804(p13.33)/chr12:27,766,831(p11.23)	chr12:405,805/chr12:27,766,847	1 + 16	KDM5A-PPFIBP1 fusion	NA	—	Continued
19	AA	—	46,XN,t(2; 17) (p13; p11.2)mat	chr2:153,997,621(q23.3)/chr17:74,377,274(q25.1)|chr2:64,928,616(p14)/chr17:6,282,825(p13.2)	Insufficient DNA specimen		SPHK1	NA	—	Continued
20	HRT21	—	46,XN,t(13; 17) (q22; q25)pat	chr13:74,775,445(q22.1)/chr17:76,814,582(q25.3)	chr13:74,775,445/chr17:76,814,582	0	USP	NA	—	Continued
21	AMA	—	46,XN,t(7; 8) (p13; p23.1)mat	chr7:45,147,959(p13)/chr8:2,511,714(p23.2)	chr7:45,147,959/chr8:2,511,718, inserted 7bp in breakpoint	4	TBRG4	NA	—	Continued

AA, autosomal aneuploidies; AMA, advanced maternal age; ARH, adverse reproductive history, HRT18 high-risk for trisomy 18, HRT21 high-risk for trisomy 21, NMR N-masked regions, NA, not available; ND, not detected; SCA, sex chromosome aneuploidy. Symbol “-” represents a negative result.

### Whole-genome sequencing coverage statistics and data analysis

The average and median depth coverage of WGS was 33 ×, and the percentage of coverage over 10 × and 20 × was 88% and 97%, respectively. Five balanced chromosome translocations occurred in centromeric or pericentromeric heterochromatic regions (due to low quality base calls with masked substitutes “N”s, i.e., undetermined bases, as known as N-masked) were not detected by WGS. WGS detected BCAs in 13 cases out of the remaining 16 fetuses, including 11 balanced chromosome translocations and two inversions. (Table 1)., WGS refined the sub-bands of chromosomal variants detected by karyotyping in the 14 cases, including two inversions. (Table 1). Sanger sequencing confirmed all, except two cases due to insufficient DNA specimen, breakpoints detected by WGS. It should be noted that, in case 21, insertion of 7bp at the breakpoint was found only by Sanger sequencing (Supplementary Figure S1), which casts doubt on the stringency of the definition of balanced translocation actually found clinically. Unfortunately, due to insufficient DNA samples from the parents, Sanger sequencing could not be added to confirm whether the insertion was inherited from the mother or *de novo*. In summary, no genomic imbalance was detected by WGS analysis in all the subjects.

Thirteen genes were found disrupted, in which the *CACNA1E* (in case 12) and *STARD7* (in case 17) genes are associated with the developmental and epileptic encephalopathy (MIM: #618285) and the familial adult myoclonic epilepsy (MIM: #607876), respectively. In addition, TADs were found interrupted in six cases. (Table 1).

### Sanger sequencing confirmation

Except for two case of insufficient DNA specimen, Sanger sequencing was performed in 11 subjects (Supplementary Figure S1). Compared with WGS results, Sanger sequencing verified that four breakpoints were identical and six with differences within 10 bp. Only one breakpoint, in case 18, showed difference in 16 base pairs. (Table 1; Supplementary Figure S1).

### Case 12

**FIGURE 2:**
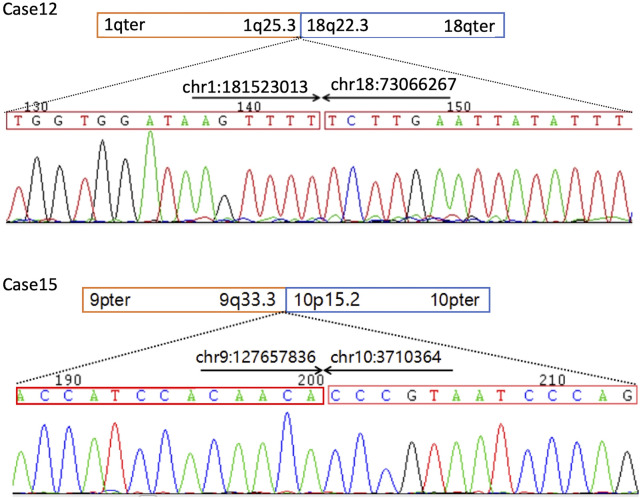
Sanger sequencing confirmation of the balanced chromosomal abnormalities (BCAs) breakpoints in Case 12 and 16. The comparison of sequences detected by WGS and Sanger sequencing in the cases is listed in Table 1. PCR primers used for Sanger sequencing as following: Case 12 F-TGGGAGGGATAAAGCGACTTG, R-CCTCCCATGACATGTAGGGATTAT, case 16 F-AACAGACTCTCCACAACAGAGAAC, R-ACTCGCCCTCCGAGTGAAAGGAG.

Case 12 was a 27-year-old female at 18-weeks pregnant, who had one previous abortion (not for medical reasons). In the first trimester of this gestation, the woman had no history of exposure to toxic substances and inappropriate medication. The pregnant woman and her husband were healthy and they had no family history. At 22 weeks pregnant, ultrasound showed her fetus had significant macrocephaly with bilateral cerebral ventriculomegaly (12/12 mm), bilateral talipes calcaneovalgus, and clenching hand. (Figure 1A). By amniocentesis, karyotyping showed the fetus had *de novo* 46,XN,t(1; 18) (q25; q23)dn (Figure 1B), while the chromosome microarray analysis (CMA) was negative, thereby singleton WGS was performed and the result showed the balanced translocation with breakpoints at chr1:181, 523, 009 (q25.3) and chr18:73, 066, 267 (q22.3) that confirmed by Sanger sequencing, chr1:181, 523, 013 and chr18:73, 066, 267, respectively. (Figure 2).

Breakpoints chr1:181, 523, 009 or 181, 523, 013 locates on intron 3 of *CACNA1E* and the translocation caused a deletion of the downstream exons in the *CACNA1E* gene. *CACNA1E*-associated epileptic encephalopathy 69 (MIM: #618285) is an autosomal dominant inherited disease characterized by inborn errors as macrocephaly and joint contractures, which matches the ultrasound findings in the fetus. According to the diagnosis, the couple chose to terminate their pregnancy at 22 + weeks gestation. The terminated fetus appeared severely bent and deformed limbs and, however, the couple did not authorize the biopsy.

### Case 15

The woman was a balanced translocation carrier and she had 46,XN,t(9; 10) (q32; p13)mat detected by karyotyping. Her first baby had congenital cranial hypoplasia, ventricular septal defect (VSD), and ASD, who died at age 8 months. In this pregnancy, amniotic fluid karyotyping showed the fetus had 46,XN,t(9; 10) (q32; p13)mat and no significant CNV was found by the previous CMA analysis. WGS reveal the translocation breakpoints at chr9:127, 657, 832 and chr10:3,710,364 that both locate on chromosomal regions without a known functional annotation (Figure 2). The couple were healthy and had no history of harmful exposure and family history. Based on the description of signs in her first baby, it is rational to speculate the previous baby with 9q34.3 terminal/subtelomeric deletion syndrome, or Kleefstra syndrome 1 (KS1, MIM: # 610,253), caused by heterozygous *EHMT1* defect, however, the previous results of genetic tests in her first baby were not retrievable. The core phenotype of KS1, including brachycephaly or microcephaly, conotruncal heart defects, severe neurodevelopmental, and behavioral impairment, explains the findings in the first baby of the woman. However, none of the signs of KS1 were found in the second gestation tests and it could not be explained by the balanced translocation. Based on the above findings, the balanced translocation was considered benign.

After genetic counseling, the couple decided to continue the pregnancy and their boy was normal at age two years.

### Case 17

Case 17 was enrolled because the prenatal screening in her second trimester revealed a high risk of Down syndrome. Both the husband and wife were healthy and had no family history of genetic disease (but could not recall whether a family member had transient benign epilepsy). This was the first child of the couple, and there was no history of exposure to toxic substances or drugs in the first trimester of pregnancy. At 20 weeks of gestation, her routine prenatal testing was normal; prenatal screening by the quad test revealed a possible high risk of Down syndrome. Karyotype analysis showed no abnormalities in chromosome number but paternal inherited balanced translocation of chromosomes 2 and 21; fetal amniotic fluid WGS showed that one of the breakpoints of this balanced translocation was located within the *STARD7* gene (Table 1), which lead to a heterozygous deletion of the last exon. *STARD7* pathogenic variants cause an autosomal dominant familial adult myoclonic epilepsy (MIM: #607876), which is characterized as epilepsy that can occur as early as puberty, but no other central nervous systemic abnormality or phenotype have been reported in infancy (De Fusco et al., 2014). However, in all reports of *STARD7*-related epilepsy, all cases are due to the expansion of the 5 bp repeat in intron 1, and other *STARD7* pathogenic variants have never been reported in patients(Corbett et al., 2019). After informing the couple of the test results, the couple decided to continue the pregnancy.

## Discussion

We demonstrated WGS being an efficient method for breakpoint mapping with a high resolution in detecting and interpreting BCAs compared to sequential and multiple tests commonly performed clinically. However, it does not always permit a phenotype correlation in *de novo* BCAs carriers while the breakpoints do not disrupt known disease-causative genes. In addition, a short-read, paired-end sequencing method cannot detect breakpoints of low-level mosaicism, Robertsonian translocations, small supernumerary marker chromosomes and ones in repetitive sequences cannot be detected (Hochstenbach et al., 2021). It is estimated that 7.6% of breakpoints were localized within genomic segments that cannot be confidently mapped by short-read sequencing (Redin et al., 2017). In this study, translocation breakpoints of 13 BCAs, including all inversions (2/2), were identified by WGS, in which 10 breakpoints were confirmed by Sanger sequencing with differences of <10 bp (Table 1). In the 12 BCAs, two cases (case 12 and 17) were identified interrupting known disease-causative genes (*CACNA1E* and *STARD7*, respectively). Eight (case 7, 11, 15, 16, 18, 19, 20, and 21) with genes disrupted by breakpoints were classified as benign or likely benign according to the ACMG guidelines. However, as an interpretation tool for large fragment variants, the ACMG guidelines for CNVs are difficult to be used to determine breakpoints (Brandt et al., 2020; Riggs et al., 2020), especially large fragment genetic variants that may be caused by intragenic breakpoints. So, we, by reviewing the protein function of these eight genes, found a potential association between PDCL defect and the phenotype in case 11. TAD disruption was found in six cases, case 14, 7, 11, 12, 16, and 17. For the three cases with negative results, case 4–6, we speculated that this may be due to the limits of the short-read sequencing, resulting in insufficient coverage depth of these breakpoint regions. For some balanced translocation breakpoints that are highly suspected of being pathogenic, we suggest additional sequencing methods for further confirmation, such as high-throughput sequencing based on a long-read method.

The results of WGS led to improved clinical utility by giving a definitive molecular diagnosis in two cases. In case 12, the diagnosis of *CACNA1E*-associated epileptic encephalopathy 69 (MIM: #618285) by WGS analysis explained the abnormalities by ultrasonography screening. In another converse situation, case 15, for example, WGS analysis of the breakpoints of 46,XN,t(9; 10) (q32; p13)mat was key to differentially diagnose with 9q34.3 terminal/subtelomeric deletion syndrome, the putative cause of a severe disease in the previous died child of the family. The results of case 15 show that in addition to the detection of candidate causative genes/chromosomes, WGS is ideal for excluding suspicious findings, especially in scenario applications of genetic counseling and family decision-making.

Phenotype-association of most of the interrupted genes are unknown, however, *PDCL* found in case 11 with situs inversus piqued our interest. Human phosducin-like protein (PDCL) is a chaperone of heterotrimeric G proteins and it, together with phosducin, a phosphoprotein, have been demonstrated to regulate G-protein signaling. (Thibault et al., 1999; Lukov et al., 2005). As far as we know, *PDCL* variants or functional defects were never found in patients with Mendelian disorders, however, in mice, Pdcl plays a role in regulating ciliary function by being a positive regulator of hedgehog signaling (Pusapati et al., 2018). Situs inversus is quite common in patients with ciliary abnormalities in human (Pennekamp et al., 2015) and *PDCL* defects found in this study may provide clues for future discovery of the new disease-causing gene.

Another particular concern is case 2, which was reported with a *de novo* TAD interruption on 4q35 (Table 1; Supplementary Figure S1), causative for Facio-Scapulo Humeral dystrophy (FSHD) (Sarfarazi et al., 1992; Lemmers et al., 2010). FSHD is characterized as adult- (90%) or childhood-onset (10%) progressive skeletal muscle disorder with a highly variable phenotype (van den Boogaard et al., 2016; Johnson and Ankala, 2020; Schatzl et al., 2021), which is not possibly detected by ultrasound screening in fetus. TADs are fundamental units of three-dimensional (3D) nuclear organization, at the sub-megabase scale, chromatin domains with high interaction frequency and relatively isolated from neighbor regions (TAD boundaries) form TADs (Lieberman-Aiden et al., 2009; Dixon et al., 2016). More recently, human development and diseases due to TAD disruptions received attention from researchers (Tena and Santos-Pereira, 2021). However, we have limited resources and framework for integrating the TADs and standard guidelines and tools for TAD analysis, for now, are not available. Therefore, long-term follow-ups, especially in case 2, are needed.

Although it is now difficult to reach a definitive diagnosis in the prenatal setting, a negative WGS result can also benefit genetic counseling by giving additional reassurance. In this study, WGS results reduced anxiety of the family found high-risk for trisomy or chromosomal aneuploidies by previous screening when interpreting BCA findings.

There are limitations to this study. Firstly, prenatal or postnatal follow-up was incomplete in most cases. This may be because these families that chose to continue the pregnancy after genetic counseling have normal newborn children, or because the WGS breakpoint suggests an association with a late-onset disease with mild phenotype, such as in case 17. In addition, integrating disruptions of TADs is still a big challenge. Secondly, cases with inherited BCAs were not excluded in this study, in which a prenatal diagnosis was considered to be made based on family history. Thirdly, the sample size of this study is small and only one with significant ultrasound abnormality. Considering of a few previously published articles using short-read WGS in prenatal studies (Redin et al., 2017; Yu et al., 2020), our study provides useful information to assess the application of WGS in prenatal diagnosis in fetuses with BCAs. Finally, it is reported that low-pass (one-fold coverage) WGS has a certain robustness for the detection of BCA (Liang et al., 2017; Dong et al., 2018), however, in the setting of clinical prenatal diagnosis, the comparison of 30X and low-pass WGS for BCA breakpoint detection is still lacking in larger studies. We hope to conduct cohort studies of these two WGS techniques in more prenatal diagnosis cases of BCA in the future, so as to discuss whether there is consistent reliability while reducing testing costs.

## Conclusion

This study demonstrated the advantages of WGS over prenatal diagnosis on the detection of BCAs. The strategy of BCA breakpoints detecting is available by WGS, which gives additional information for prenatal diagnosis. Although, for now, only the case with abnormality revealed by routine prenatal screening reached a definitive diagnosis, WGS results give physicians potential opportunities to improve the genetic counseling and perinatal care, and further reduced anxiety in the families coming for medication.

## Data Availability

The data presented in the study are uploaded in the Supplementary files, accessible via the link: https://www.frontiersin.org/articles/10.3389/fgene.2022.951829/full#supplementary-material.
