# Supplementary material for: Prenatal diagnosis of 21 fetuses with balanced chromosomal abnormalities (BCAs) using whole-genome sequencing
Source: Front Genet. 2022 Sep 15;13:951829. doi: 10.3389/fgene.2022.951829 (PMC9520355; doi:10.3389/fgene.2022.951829)
Supplement: Supplementary file 1 [file Table2.DOCX]

Supplementary Material


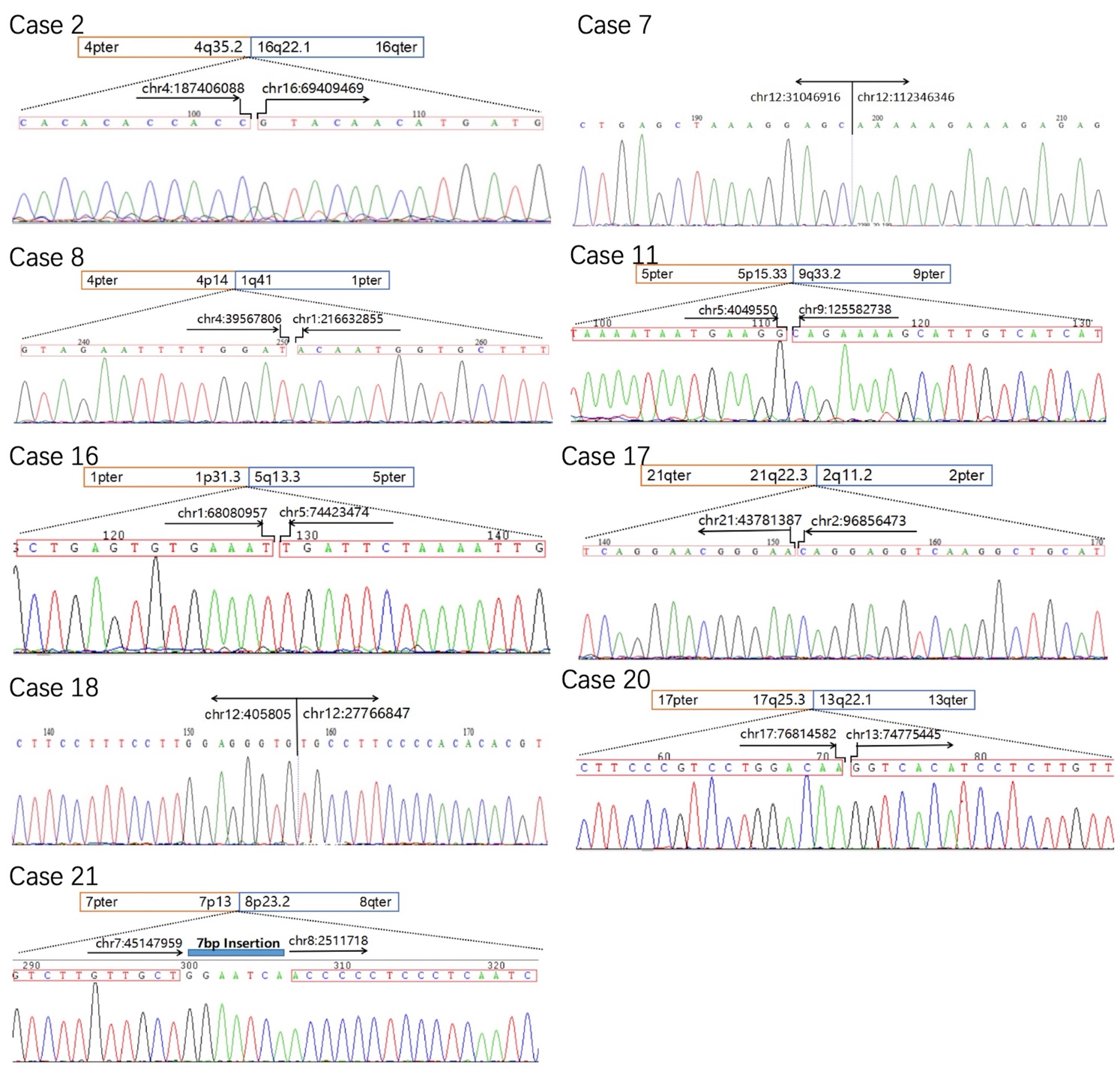


**Supplementary Figure 1.** Sanger sequencing confirming the breakpoints of balanced chromosomal abnormalities (BCAs) detected by whole-genome sequencing (WGS) in nine cases. The comparison of sequences detected by WGS and Sanger sequencing in the cases is listed in Table 1.

PCR primers used for Sanger sequencing: Case 2 F-AGAAGCTGAAACAAAATTAATGTAGTA, R- AGATGTGAAGGCTATTCAATGGGA, case 7 F-CAAAAGCCCATCTCTAGCTC, R-GCATCTACTGAAATGACCGTA, case 8 F-CCCAACCCCTGAATTCTACTCCT, R-CCTGCACAAGCTCTCTTCTCTTG, case 12 F-CGCTGGCATTTCAAAGATGTCATA, R-CAACCATTCATGGCTTCGGTC, case 17 F-TCAGGTAGTATGCGAGGCCAT, R-GAGACATCCTAAAAGTACAGGACAG, case 18 F-CTTCTGGGGTCATGTCAAAGGGGCTT, R-TCTCTGCAGCCAGACGGTTCCAGG, case 19 F-TGGCTGCCTTATATAAATAACTGGT, R-CCAGCACGAGGGACTTTCTT, case 21 F-TGGGATAAACCCAGAAACGTC, R-TACATTTGCTTAACCAAGGAGAA, case 22 F-GAATCAGCTGGCCACATCCCACT, R-TTAGAGGAGACCATCACTCTCACCA
